# Perspectives of Family Physicians on Computer-assisted Health-risk Assessments

**DOI:** 10.2196/jmir.1260

**Published:** 2010-05-07

**Authors:** Farah Ahmad, Harvey A Skinner, Donna E Stewart, Wendy Levinson

**Affiliations:** ^4^Department of MedicineUniversity of TorontoTorontoCanada; ^3^Women's Health ProgramUniversity Health NetworkUniversity of TorontoTorontoCanada; ^2^Faculty of HealthYork UniversityTorontoCanada; ^1^Social and Behavioral Health Sciences DivisionDalla Lana School of Public HealthUniversity of TorontoTorontoCanada

**Keywords:** Computer, health, risk assessment, screening, psychosocial, primary care

## Abstract

**Background:**

The firsthand experience of physicians using computer-assisted health-risk assessment is salient for designing practical eHealth solutions.

**Objective:**

The aim of this study was to enhance understanding about computer-assisted health-risk assessments from physicians’ perspectives after completion of a trial at a Canadian, urban, multi-doctor, hospital-affiliated family practice clinic.

**Methods:**

A qualitative approach of face-to-face, in-depth, semi-structured interviews was used. All interviews were audio recorded and field notes taken. Analytic induction and constant comparative techniques were used for coding and analyses. Interpretation was facilitated by peer audit and insights gained from the social exchange theoretical perspective.

**Results:**

Ten physicians (seven female and three male) participated in the interviews. Three overarching themes emerged in relation to computer-assisted health-risk assessments: (1) perceived benefits, (2) perceived concerns or challenges, and (3) feasibility. Physicians unanimously acknowledged the potential of computer-assisted health-risk assessments to open dialogue on psychosocial health risks. They also appreciated the general facilitative roles of the tool, such as improving time-efficiency by asking questions on health risks prior to the consultation and triggering patients’ self-reflections on the risks. However, in the context of ongoing physician-patient relationships, physicians expressed concerns about the impact of the computer-assisted health-risk assessment tool on visit time, patient readiness to talk about psychosocial issues when the purpose of the visit was different, and the suitability of such risk assessment for all visits to detect new risk information. In terms of feasibility, physicians displayed general acceptance of the risk assessment tool but considered it most feasible for periodic health exams and follow-up visits based on their perceived concerns or challenges and the resources needed to implement such programs. These included clinic level (staff training, space, confidentiality) and organizational level (time, commitment and finances) support.

**Conclusions:**

Participants perceived computer-assisted health-risk assessment as a useful tool in family practice, particularly for identifying psychosocial issues. Physicians displayed a general acceptance of the computer tool and indicated its greater feasibility for periodic health exams and follow-up visits than all visits. Future physician training on psychosocial issues should address physicians’ concerns by emphasizing the varying forms of “clinical success” for the management of chronic psychosocial issues. Future research is needed to examine the best ways to implement this program in diverse clinical settings and patient populations.

**Trial Registration:**

ClinicalTrials.gov  NCT00385034; http://clinicaltrials.gov/ct2/show/NCT00385034 (Archived by WebCite at http://www.webcitation.org/5pV8AGRgt)

## Introduction

In today’s era of eHealth technologies, interactive computer applications are transforming medical practice and empowering health consumers [1,2]. Some of these applications focus on patients to provide them with information, social support, and training in coping skills (eg, Internet kiosks and networking websites), while others focus on clinicians to improve the consistency and quality of care they provide (eg, handheld digital devices with decision-trees on differential diagnosis and treatments). However, the utility of eHealth tools is not limited to use only by patients or providers The recent wave of eHealth innovations attempts to connect patients and clinicians, benefiting both simultaneously. One such example is the computer-assisted health-risk assessment (HRA) where patients complete a computer survey before seeing their clinician. The interactive program then prints an individualized risk report for the clinician and a recommendation sheet for the patient just before the medical consultation. The intention of such computer-assisted health-risk assessment is to facilitate face-to-face consultation with the provider and not to substitute for patient self-care. Computer-assisted health-risk assessments have many advantages including increasing time efficiency, response accuracy, and providing tailored questioning with skip patterns (eg, not asking how many cigarettes one smokes if a respondent is nonsmoker) [3,4]. Further, studies have demonstrated patients’ positive attitudes toward its use [5].

Recently, Rhodes et al [6-8] and the authors [9] studied the effectiveness of a multi-risk computer-assisted HRA tool. In these studies, the interactive computer survey was completed by patients using touch-screen technology and included questions on psychosocial risks (alcohol, tobacco and street drug use, sexual health, conflict in relationships, and depression), road and home safety, cardiovascular risks, and sociodemographics. The recommendation sheet for patients provided them with simple language health suggestions and contact numbers of relevant community services. The one-page risk report for physicians indicated the patient’s positive responses to the health-risk questions and the community services to which the patient could be referred for the reported risks. These studies, which were randomized trials [6-9], were conduced in an emergency department [6-8] and in family practice [9] settings. The results suggested that computer-assisted HRA improved patient disclosure and physician detection of the risk of partner violence and compromised mental health. For example, in the study conducted in an urban emergency department, the rates of provider detection of partner violence were 14% in the patient group that had completed a computerized screening questionnaire versus 8% in the usual care group (*P*= .07) [8]. In the family practice setting, provider detection of partner violence occurred in 18% of the computer-screened patient group compared with 9% of the usual care group (adjusted relative risk, 2.0; 95% confidence interval [CI] 0.9 - 4.1) [9]. In the same study, provider detection of compromised mental health occurred in 36% of the patients in the computer-screened group compared with 25% of the usual care group (adjusted relative risk, 1.5; 95% CI 1.0 - 2.2).

Thus, computer-assisted health risk assessments provide a positive and salient change in clinical practice because both partner violence and compromised mental health issues remain under detected in routine medical visits [10-17] despite their seriousness and high prevalence [18,19]. Wider incorporation of computer-assisted HRA could facilitate the orientation of health services toward a comprehensive concept of health and well-being. However, the “real life” success of any intervention is contingent upon its acceptance by users and its contextual feasibility.

The aim of this study was to enhance understanding of the attitudes of family physicians toward a computer-assisted HRA after they had used this tool in a randomized controlled trial conducted by the authors [9]. The study site was a multi disciplinary family practice clinic affiliated with a teaching hospital in the inner city of Toronto.

## Methods

### Study Design

We used a qualitative research approach to develop in-depth understanding about perspectives of physicians [20,21]. Face-to-face, semistructured, in-depth interviews were conducted with family physicians to elicit their perceptions of and experiences with the computer-assisted HRA. As all potential participants worked at the same clinic, individual interviews were preferred over focus groups. This resulted in scheduling of the interviews at times convenient to each participant and ensured participants’ confidentiality [22,23]. Physicians were eligible to participate if they had seen at least five patients who had participated in the randomized trial. This purposeful sampling allowed information-richness in relation to the studied phenomenon [24,25]. For an exploratory study with a homogeneous sample, five to eight participants are generally considered sufficient [26,27]. We considered our sample homogeneous because all participants were physicians working at the same clinic, and all had used the computer-assisted health-risk assessment tool. The study procedures were approved by the ethics review boards of the University of Toronto and the hospital with which the family practice clinic where the study took place was affiliated.

### Participants

Ten eligible physicians (seven females and three males) participated in the interviews which were conducted between October and November of 2005. The average age of participants was 46 years (range 32-64 years). Participants had been in practice for 16 years on average (range 1-30 years) and reported practicing 30 to 50 hours per week. At the trial site, physicians’ weekly number of hours ranged from 16 to 40. Eight of the physicians reported seeing female patients at 50% or more of visits.

### Data Collection

All interviews were conducted by the first author at a place and time convenient to the physician.

Participant physicians provided written consent and completed a one-page demographic questionnaire before the interview. No monetary incentives were offered. The interviewer used the principle of “ask, wait, and probe” and a semistructured interview guide with open-ended questions [23]. All interviews were audio recorded and field notes were taken.

The interview guide was constructed jointly by the research team and clinical collaborators (a family physician, a nurse, and a social worker) from the study site. This was informed by our literature review on the modes of inquiry for psychosocial health risks [28-33]. We identified dual barriers for the face-to-face encounter of physicians and patients. Patient barriers included feelings of embarrassment, fear of physician’s rejection or reaction, concerns about confidentiality, and lack of physician’s direct inquiry [34-36]. Physician barriers included discomfort, fear of patient’s negative reactions, lack of time, priority of the acute problem, and lack of familiarity with resources [10-13]. At the same time, computer-assisted HRA was identified as having potential to address many of these barriers on the patient (eg, desire of “direct questioning” by provider in a nonjudgemental manner) and provider side (eg, time efficiency, referral information, and anonymity). This informed the development of topic areas and probes for the interview guide (eg, sensitivity of certain health risks and visit time).

The use of open-ended questions and probes in the guide allowed defining the research area without restraining the expressions of participants [22,23]. The guide was revised after the first two interviews and included four key questions: (1) What do you think of your experience with the computer-assisted HRA? (2) How would you describe its potential across various risks and visits? (3) Would you recommend such computer-assisted HRA in a family practice setting? and (4) What factors are important for its implementation in a family practice setting?

### Data Management and Analysis

The interviews with the physicians were taped, transcribed verbatim, and from the transcriptions, Word files were prepared. The techniques of analytic induction and constant comparison were used to code and analyze the transcripts [37]. Analytic induction, originating from Znaniecki’s work [38], entails the development of concepts and testing of propositions from the data by a systematic examination of the similarities between various social phenomena (eg, acceptance or rejection) and processes (eg, agent-agent and agent-structure relations) while emphasizing the research context and negative cases that challenge an emerging finding and thus lead to analytical refinement [39,40]. Also, the analytical method of constant comparison, derived from the grounded theory approach [41], was used within and between interviews [42]. Informant statements were compared for thematic and/or conceptual similarities within and between interviews (eg, What do these quotes have in common? What is unique?)

Coding involved collating and analyzing all of the transcribed data related to the emerging themes and concepts informed by our literature review. An initial coding scheme was developed jointly by the research team after all members had read the collected information [43,44]. This initial coding scheme was then applied to the text data by assigning symbols to represent each category. The sorted and coded data was then read and read again by the first and last author to refine the analysis and to control researcher bias [37]. For rigor, attention was directed to the range and diversity of experiences, meanings, and perceptions along with a search for disconfirming evidence. For example: What does this tell about how participants developed their perspectives? How do these relate to each other? How are emerging relationships confirmed or disconfirmed within data?

All participating physicians emphasized the on going nature of their relationship with patients. This prefaced both positive and negative perceptions of physicians with respect to the computer-assisted HRA tool. For interpretation of this two-sided aspect of the tool, we used the lens of social exchange theory [45,46]. This theory has been applied at both micro and macro levels to explore the provider-patient relationship [47-49]. According to this theory, health services are provided through an exchange process in the physician-patient relationship. Patients need the physician’s knowledge and competence to restore or maintain their health. Physicians need patients for income, new patient referrals, and the less tangible rewards of compliance, praise, and appreciation. Patients’ relative dependency on physicians is influenced by the degree of their trust in physicians while increasing trust in return enhances the social responsibility of physicians. Limited successes in social exchanges give rise to negative emotions (eg, self-depreciation, guilt, anger, or discomfort with others), which act as internal stimuli to avoid recurrence of these emotions. Therefore, a tool which can change the nature of social exchange between physician and patient and/or physician and institution, such as computer-assisted HRA, can be perceived as helpful or not helpful depending on how it influences the exchange and provokes negative or positive emotions. The inclusion of the theoretical lens (ie, social exchange theory in this study) at the time of interpretation augments rigor and trustworthiness of the findings [50]. We also incorporated the technique of peer audit by sharing our findings with the site collaborators whose feedback refined our interpretation, adding credibility [37,50].

## Results

**Figure 1:**
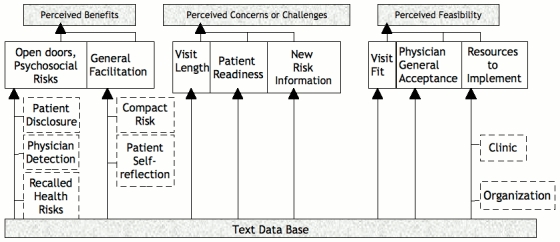
Physician in-depth interviews: themes, subthemes, and subcategories

Three overarching themes (Figure 1) emerged in relation to computer-assisted HRA. These were: (1) perceived benefits with subthemes of opening doors for psychosocial risks and general facilitation; (2) perceived concerns or challenges with subthemes of new risk information, patient readiness, and visit length; and (3) feasibility with subthemes of general acceptance, visit fit, and resources. For each theme, we present dominant views as well as provocative dissenting views, where applicable. Representative quotations from the data exemplifying each theme and subtheme are presented in Tables 1 to 3.

### Theme 1: Perceived Benefits

Benefits of the computer-assisted HRA emerged as a dominant theme across all physician interviews and included two subthemes. The tool was unanimously perceived to open dialogue on psychosocial health risks. The HRA or screening was also perceived to serve a general facilitative role in providing patient care in a busy clinic environment. We discuss each subtheme and its subcategories (also see Table 1).

#### Subtheme: Opening Doors for Psychosocial Risks

All physicians agreed with the potential of the computer-assisted HRA for “opening doors” for discussion on socially sensitive health risks. Physicians’ comprehensive discussions on this sub-theme included three subcategories: types of risks recalled, mechanism of patient disclosure, and mechanism of physician detection.

Physicians recalled having discussions on multiple socially sensitive issues due to the computer generated risk reports. These included risks of partner abuse, depression, safe sex practices, use of street drugs, and alcohol overuse. Physicians discussed the various mechanisms in which the computer-assisted HRA possibly improved patient disclosure and physician detection of the psychosocial risks. They attributed enhanced patient disclosure to the tool characteristics in that it asked specific questions, gave permission to talk, and provided an anonymous and unrushed mode of disclosure. One physician drew a metaphoric similarity between the computer-assisted HRA and a brochure on domestic assault as message conveyers about the readiness of family physicians to address the issue. Physicians attributed their detection of new psychosocial risks to the comprehensiveness of the computer survey while acknowledging they often miss these risks. Some physicians commented that the computer-printed report helped them to initiate conversations on socially sensitive risks in a straightforward manner.

**Table 1:** Physician perceived benefits of the computer-assisted HRA

Subtheme and Subcategories		Representative Quotations
**Opening doors for psychosocial risks**		
	Recalled health risks	I think screening around issues like mood, depression, and abuse, I think it could be really, really good for that. (Interview # 2, page 4)
	Patient disclosure	Often it gives permission that patients might not even answer it correctly initially, but it might open up dialogue in the future. (Interview # 8, page 5)
		When things are a little bit anonymous, I think that people, if they’re a bit shy or reticent, will come out with more, particularly if the interview is rushed. I think that’s a problem here. (Interview # 7, page 2)
	Patient disclosure	Patients don’t necessarily think they’re medical. I’ve had a patient who was raped who said to me, “I wasn’t sure if I should tell you about this because I wasn’t sure if it was a medical problem. Do you deal with this?” And you know, obviously that’s a message you want to get out there, is that yes, we do deal with this... So, absolutely any information is good! And I think the reason she did that is that I had a message on the wall about domestic violence. (Interview # 4, page 3)
	Physician detection	It allows you to be more comprehensive; or at the very least, allows you to identify things that sometimes in a physical setting or in an appointment, you don’t have time to get to. (Interview # 2, page 4)
		Um, well I guess it just gives a starting point to the discussion that you know, “you said here that you used marijuana in the past” and just sort of acknowledging it and then, “how much are you using?” It’s just sort of a good starting point. And asking them what they thought of the survey. Was there anything that they learned from the survey? And then they might bring it up. (Interview # 10, page 2)
**General facilitation**		
	Compact risk report	Because it was very compact. So, you got a lot of information right in front of you, without obviously having to ask about all of it. So, you could hone in on the things that needed to be dealt with and that was nice. (Interview # 7, page 2)
	Patient self reflection	[When completing computer survey] in the privacy of their own room or waiting room or whatever, they could sit and think about it. And they could change their minds. There is that sort of time for reflection. (Interview # 4, page 5)
		In some instances it made patients aware of problems that they weren’t—that were sort of at the back of their minds, that they weren’t really aware of. (Interview # 7, page 1)

#### Subtheme: General Facilitation

Physicians also discussed the general facilitative roles of the computer HRA, particularly its compact time-efficient nature and patients’ self-reflections.

Physicians found the one-page risk summary generated from the more comprehensive review especially useful and, in their words, “compact.” The one-page summary was said to have saved the physician time to screen for health risks.

Many physicians remarked that the computer survey seemed to enhance patients’ self-reflection by asking about several health risks in a private and unrushed manner. They linked patients’ heightened self appraisal to their improved risk recognition. Further, most of the physicians described patient reactions as positive, using expressions such as “showed interest,” “felt comfortable,” “felt more cared for,” “analyzed reports [with me],” “wished to be in the computer group,” “happy,” and “seemed to [have] had benefit from it.”

### Theme 2: Perceived Concerns or Challenges

Physicians discussed some concerns or challenges in relation to the computer-assisted HRA. Three subthemes emerged: scope of new risk information, patient readiness, and length of visit. Participants expressed both positive and negative stances (Table 2).

**Table 2:** Physician perceived concerns/challenges of the computer-assisted HRA

Subtheme and Divergent Views		Representative Quotations
**New risk information**		
	Positive stance	Um, it was interesting and in terms of sometimes bringing up topics that wouldn’t have normally come up. Because sometimes that happens in family medicine that you know your patients so well that you don’t necessarily go over the same old ground every visit. And so it would actually bring these things up in a timely manner. (Interview # 4, page 1)
	Negative stance	I didn’t have any problem with it. It didn’t really give me any new information that I didn’t already know about my patients...Now it would be very different in a department like emergency where they don’t have that ongoing relationship. Or for a busy physician who perhaps doesn’t talk about psychosocial issues. (Interview # 8, page 1)
**Patient readiness**		
	Positive stance	Some of the things were actually quite, um, quite different in terms of why the patient came in, in terms of what the survey picked up. And so a lot of the time we would acknowledge it and then ask the patient, you know, “did you want to focus on this, or focus on the primary reason” they came in. (Interview # 6, page 1)
	Negative stance	To do it when somebody comes in for a sore throat, or blood pressure…I don’t know that that would be the best timing. Mind you, the best timing is, when the patient is ready. (Interview # 9, page 2)
**Visit length**		
	Positive stance	[When] they were in here to discuss their high blood pressure and their diabetes, and there’re no other issues around what we’ve been [given]—the computer survey generated—I did not push it at that point…You’d ask about it, but then say, well maybe you should come back about that. That’s what you’d have to do. Because if they’re in and out and there are five people waiting, it’s not good. I’d probably put it in my notes…to discuss. (Interview # 7, page 4)
	Negative stance	There were all these issues that were brought to light, but most of them were over…it did lead to more time with the patient of course…a lot of them were over diagnosis. (Interview # 10, page 1)

#### Subtheme: New Risk Information

In the randomized controlled trial, eligible patients were recruited without differentiating the purpose of their visit (new patients were not recruited). Some physicians felt that computer-assisted HRA had limited use in generating new risk information for most of their patients because they had seen them for several years and knew their risk profile. In contrast, some physicians shared their surprised detections. One physician was critical of the notion of “knowing the patient” in family practice and emphasized the potential of the patient risk profile to change overtime. This physician remarked, “It would actually bring these things up in a more timely manner.”

#### Subtheme: Patient Readiness

A few physicians expressed concern about the readiness of patients to discuss the risks which may have been identified by the computer assessment when they were unrelated to the main reason for their visit to the clinic. For example, they remarked that patients coming for acute health problems, such as high fever, may not feel comfortable discussing the computer-reported psychosocial issues.

However, other physicians emphasized the various ways to manage patient hesitation. They discussed the possibilities of noting the patient-reported risks in the chart, offering follow-up visits, or inquiring about a patient’s wish to talk about the reported risk in that particular visit. Comments from these physicians reflected a positive stance as they considered the potential benefits of computer-generated risk reports across many types of visits in the context of ongoing care in family practice.

#### Subtheme: Visit Length

Contact time with the physician during the health care visits of the trial patients varied from brief (eg, acute care visit) to lengthy (eg, periodic health exam). A few physicians expressed concern about the increase in length of the visit due to the additional task of reviewing the computer generated risk report.

In contrast, other physicians described managing the time pressure by offering follow-up visits or viewed the task of risk review as a professional obligation even if it meant increases in the consultation time. They explained using the option of a follow-up visit in order to avoid “taking time away” from other waiting patients, mirroring the individual- versus collective-responsibility dilemma, discussed below.

### Theme 3: Feasibility

The overall feasibility of the computer-assisted HRA in a family practice setting emerged in physician discussions. Three subthemes characterized concerns about feasibility: general acceptance of the tool; the tool’s fit with the visit; and availability of clinical and organizational resources for its implementation (Table 3).

#### Subtheme: General Acceptance

Physicians accepted the patient administered computer-assisted HRA with varying intensity. Two of the ten participant physicians (20% of sample) were highly enthusiastic about computer-assisted HRA and perceived it as useful for all types of visits. Six of the participant physicians (60% of sample) had moderate acceptance and wished to have more details about the tool’s utility and the results of the trial in which they participated. Two physicians (20% of sample) expressed a conditional acceptance of the tool provided the results are prescreened by a nurse before the physician sees the patients because of the perceived onus on physician time. This pattern is consistent with the “innovation adoption curve” of Roger’s theory of diffusion of innovation [51]. This curve estimates the proportion of adopters as innovators/early adopters (17%), early/late majority (68%), and laggards (16%). Thus, 20% of the participants who were very enthusiastic seem to fit the innovator/early adopter group while 60% with moderate acceptance fit the late majority group of the Roger’s theory.

**Table 3:** Physician perceived feasibility of the computer-assisted HRA

Subtheme and Subcategories		Representative Quotations
Visit fit		I would say in the annual health exam...Otherwise we’re going to find that the patient’s coming in for something else and we only have fifteen minutes. We don’t have time to deal with it. (Interview # 8, page 6) The right time is when the patient is ready to do it. So, it could be a follow-up visit. It could be a physical. (Interview # 9, page 2)
General acceptance		Oh, absolutely. I think it’s a great idea. I think it’s really good [enthusiastic]. (Interview # 7, page 5) As far as I am concerned, if something like this is to be used as part of the screen, it’s perfectly fine [moderate]. (Interview # 1, page 3)
**Resources to Implement**		
	Clinic (patient flow)	Not interrupting patient flow that much...[l]ike if they know before physical, you have ten minutes allotted for this screen, so come ten minutes early. (Interview # 2, page 3)
		You’d have to have some allied health professional to do that [explain to patients]. (Interview # 4, page 6)
	Clinic (space privacy)	We’re so short on space, I don’t know where we would…and I don’t know that it would be fair for those patients to fill out a survey while they’re in the waiting room. They have to have a private place to do that. (Interview # 9, page 6)
	Clinic (information privacy)	How do you house that information? How do you keep that information confidential? What do you do with the information? And how that flows?...it’s something really quite sophisticated...our clinic is a little bit archaic in terms of its record keeping...the only thing, to try to fuse them both together. (Interview # 6, page 3)
	Organization (time and money)	Time, time, and time (light laugh). So, I mean the administration of something of this nature. There is a cost involved. (Interview # 1, page 4)
	Organization (amalgamation of policies and needs)	Things like addictions in this area are very common. Um, mental health is up there, quite high, in terms of depression, anxiety, abuse. You know, so anything related to the sort of top ten diagnoses. I’m part of the quality steering committee, we look at quality issues in terms of immunizations, pap tests, mammograms, cholesterol screening…so, uh, I guess from an organizational standpoint, it would be nice [to set priorities according to the local needs]. (Interview # 3, page 3)

#### Subtheme: Visit Fit

When asked about the tool’s potential, all of the physicians considered it most appropriate for the periodic health exam and/or for follow-up visits in a family practice context. However, the reasons for this recommendation varied. Many commented on the need for a fit between the preventive focus of the HRA tool and the main reason of the visit in order to overcome the issues of time constraints and/or patient readiness. Two physicians considered the tool useful across all visits but recommended its use be limited, at least in this early phase of the initiative, to periodic examinations or follow-up visits due to logistical concerns (discussed below). Two physicians also expressed interest in the HRA tool for first-time visits due to its comprehensive and time-efficient nature.

#### Subtheme: Resources to Implement

When asked about the future implementation of computer-assisted HRA, physicians discussed resources needed at the clinic and organizational levels. At the clinic level, physicians emphasized mobilizing or strengthening the resources to manage patient flow and provide private space and confidentiality to patients. For an appropriate flow of patients, physicians focused on time-efficiency and information-flow by proposing the early arrival of patients and the training of allied health staff to monitor the tool’s administration. Physicians perceived that provision of a private space for patients to complete the computer survey was important due to the sensitivity of risks included in the survey. Likewise, some physicians emphasized the need to ensure patient confidentiality. One physician acknowledged the HRA tool could be a technological challenge for “archaic” practices where computer technology has not yet been introduced

At the organizational level, many physicians emphasized time and money as necessary resources to implement computer-assisted HRA. As a solution, some proposed a model of comprehensive primary care services such as family health teams as feasible sites for implementation of the HRA tool because of the available resources within such settings. Some physicians discussed the need to amalgamate clinical guidelines, organizational priorities, and/or a local risk profile of patients to prioritize the inclusion of health risks in the computer HRA programs.

## Discussion

To our knowledge, this is the first in-depth study of physicians’ perceptions and experiences with a computer-assisted HRA program for psychosocial health risks. Participants unanimously acknowledged the potential of the computer for assessing socially sensitive psychosocial health risks. They showed general acceptance of this mode of health-risk assessment and evaluated its utility rigorously in the context of an ongoing physician-patient relationship. Participants viewed the use of computer-assisted HRA as most feasible for periodic health exams and/or follow-up visits in a family practice setting, based on perceived benefits, concerns or challenges, and logistics.

### Perceived Benefits

Perceived benefits of the computer-assisted HRA for psychosocial health risks emerged as a dominant, crosscutting theme. Physicians felt the tool improved patient disclosure and physician detection of psychosocial health risks, consistent with recent effectiveness studies [6-9]. Participants also discussed possible underlying mechanisms of this positive aspect. Physicians attributed improved patient disclosure to the tool’s specific inquiry about these risks and its anonymity in conjunction with the patients’ time for self-reflection. In other words, physicians felt that patients were empowered to disclose and discuss their socially sensitive risks with a higher level of comfort and confidence. This is in accordance with existing research on modes of inquiry [28-31] and “activated patients” [52-55], who receive pre-visit interventions (eg, education about their health risks) to become knowledgeable and willing to discuss their risk status. These activated patients become effective “prompts” for the medical providers, leading to the provision of health preventive and promoting care in medical visits. We have also conducted qualitative interviews with the participant patients to understand their enablement and empowerment; this work is in progress.

In our study, physicians attributed their enhanced detection of patients’ psychosocial risks to the consistency and comprehensiveness in risk screening provided by the tool. These findings imply that computer-assisted HRA enhanced the patient-centeredness of the physician-patient interaction [56], an area that needs further exploration. In brief, future use of the tool in a family practice setting could benefit a large number of patients seeking care for multiple health reasons.

### Perceived Concerns and Challenges

Some concerns or challenges also emerged in relation to the newness of the risk information generated by the computer program, patient readiness to discuss the reported risks, and the increase in visit time. Few physicians discussed the dilemma of providing adequate care to one versus many patients within limited time. Some physicians proactively managed these challenges by assessing a patient’s willingness to discuss the risks, taking notes, and/or setting up follow-up visits. Indeed, strategic management of the perceived barriers is possible in future applications of the tool. For example, physicians should receive comprehensive training on the varying forms of “clinical success” for the management of chronic psychosocial issues so that they don’t feel frustrated when dealing with these complex cases in the time constrains of a medical visit. The training program should emphasize various stages of patient-readiness to take an action [57,58] along with motivational interviewing techniques [59,60] and the physician’s gatekeeper role of making referrals to other services. Management of psychosocial issues often requires diverse health and social resources [61,62], and physicians need not be the sole providers of care. Also, multidisciplinary models of care and the incentive of billing codes for counseling hold potential to address physicians’ time concerns.

### Computer Mediated Visits

Physicians perceived the success of computer mediated patient interactions in light of the perceived benefits and concerns or challenges of this type of interaction. Interactions were perceived as successful when patients shared health risk information (they disclosed and were ready to talk) and when the information was new to the physicians and led to the provision of care, provided time was available. The interactions were perceived as partially successful when patients disclosed but were not ready to talk, and/or physicians did not have enough time to adequately deal with the reported risks. Based on Lawler’s social exchange theory and related research [63,64], exchanges with low success generate negative emotions, such as frustration leading to low self-efficacy. These internal stimuli in turn lead to motivations to avoid recurrence of negative feelings, consistent with the social cognitive theory [65,66]. Accordingly, physicians in our study sought to reduce the likelihood of partially successful exchanges. Because of the preventive focus of computer-assisted HRA, physicians recommended the use of this tool for periodic health exams and/or follow-up visits where time and patient-readiness were not seen as undermining factors. Also, prevention is a built-in focus of periodic health exams. It seems that system level supportive mechanisms are needed to enhance physicians’ confidence in their ability to manage psychosocial health risks [67].

### Resources to Implement

Physicians discussed the need for clinic and organization-related resources to implement the use of computer-assisted HRA in the future. At the practice level, physicians emphasized the management of patient flow through early appointments and staff training. Patient privacy and confidentiality were viewed as important for completion of the computer surveys, but lack of space and technological skills were considered logistical limitations. These findings provide practical insights for future initiatives on computer-assisted HRA in a family practice setting and have theoretical implications for advancing understanding about diffusion of innovations [51,68]. At the organization level, physicians pointed toward the need for greater financial support or mitigation of time investment. Indeed, institutional prioritization is salient for health-promotion and disease-prevention orientation in medical settings [69]. Health care service institutions could incorporate effective computer-assisted HRA tools as part of their quality initiatives because of their potential to detect socially-sensitive health risks (eg, poor mental health, partner violence, or substance abuse) in a timely and efficient manner. This could be especially beneficial in assisting vulnerable populations who are exposed to higher risks of psychosocial issues. Thus, from the population health perspective, system level adoption of such programs could play an important role in addressing health inequities.

Participant physicians also wished for the merging of clinical practice guidelines, institutional goals, and local patient needs in the identification of health risks for assessment or screening. This reflects not only physicians’ multiple roles at various levels of health care (individual, institutional, public health) but their desire to have coherence within policies. Lack of consistency in policies across health sectors is often reported as a barrier to timely screening practices [70]. There is a need to actively involve clinicians, public health experts, health care administrators, and policy makers to establish locally tailored coherent screening guidelines.

Future direction for implementation should draw from the tenets of diffusion of innovation theory [51]. For example, early efforts could focus on settings with characteristics of enthusiastic “early adopters” who use the data on an innovation to make their own careful adoption decisions. Their success then creates a domino effect where a larger majority adopts the innovation at a pace quicker than the average.

The emerging eHealth tools can contribute to new models of care by linking clinic care and self care. For example, computer-assisted HRA (augmenting clinic care) could be offered in conjunction with “virtual clinics” and “e-messaging” to patients, supporting self care [71-73]. This evolving area holds potential to improve timely access to health care with fewer errors, leading to patient empowerment and cost savings.

### Limitations

Some limitations in the design of our study warrant caution for the interpretation of the results. Participant physicians who volunteered in a randomized controlled trial of the computer-assisted HRA practiced at an inner-city, hospital-affiliated, academic family practice clinic. The views of these physicians may not represent the views of physicians practicing at different sites. The qualitative nature of the study may limit the applicability of results to wider clinical settings. We used several strategies for rigor and trustworthiness (as described in the methods section), such as a jointly agreed upon initial coding-scheme and a theoretical lens along with peer audit for the interpretation. This increases our confidence in the transferability of findings. The data were collected in 2005, but we don’t anticipate much change in the studied population. Perhaps patients today are more likely to be acquainted with computer-assisted tools in medical settings or electronic health records than they were five years ago.

### Conclusion

Participant physicians perceived computer-assisted HRA as a useful tool in family practice, particularly for the detection and discussion of psychosocial health risks. Physicians displayed a general acceptance of the computer HRA tool and indicated its greater feasibility for periodic health exams and/or follow-up visits than for all visits. Physician training on psychosocial issues should address physicians’ concerns about patient readiness and visit time by emphasizing the varying forms of “clinical success” for the management of chronic and complex psychosocial issues. Future research is needed to examine the best ways to implement this program in diverse clinical settings and patient populations. From a public health perspective, computer-based HRA in a family practice could mean timely psychosocial risk identification and access to care for many people.
